# MiR-184 Combined with STC2 Promotes Endometrial Epithelial Cell Apoptosis in Dairy Goats via RAS/RAF/MEK/ERK Pathway

**DOI:** 10.3390/genes11091052

**Published:** 2020-09-07

**Authors:** Jiuzeng Cui, Xiaorui Liu, Lichun Yang, Sicheng Che, Hongran Guo, Jincheng Han, Zhongshi Zhu, Binyun Cao, Xiaopeng An, Lei Zhang, Yuxuan Song

**Affiliations:** College of Animal Science and Technology, Northwest A&F University, Yangling, Shanxi 712100, China; 2017050438@nwafu.edu.cn (J.C.); liuxiaorui@nwafu.edu.cn (X.L.); yanglichun2018@nwafu.edu.cn (L.Y.); gd2020@nwafu.edu.cn (S.C.); ghran2020@nwafu.edu.cn (H.G.); hanjincheng@nwafu.edu.cn (J.H.); zhuzhongshi123@nwafu.edu.cn (Z.Z.); caobinyun@nwafu.edu.cn (B.C.); axpdky@nwafu.edu.cn (X.A.); zhanglei55@nwafu.edu.cn (L.Z.)

**Keywords:** miR-184, STC2, endometrial epithelial cells (EECs), apoptosis, dairy goats, receptive endometrium

## Abstract

The endometrium undergoes a series of complex changes to form a receptive endometrium (RE) that allows the embryo to be implanted. The inability to establish endometrial receptivity of livestock causes embryo implantation failure and considerable losses to animal husbandry. MicroRNAs (miRNAs) are a class of noncoding RNAs. Studies have found that miRNAs can regulate many critical physiological processes, including the establishment of RE during embryo implantation. miR-184 is highly expressed in the endometrial receptive period of dairy goats. This study aimed to explore the effect of miR-184 on endometrial epithelial cell (EEC) apoptosis and RE establishment. Stanniocalcin2 (STC2) is a direct target of miR-184, and miR-184 decreases the expression of STC2 in dairy goat EECs. miR-184 can activate EECs apoptosis through the RAS/RAF/MEK/ERK pathway. Additionally, miR-184 increases the expression levels of RE marker genes, such as forkhead box M1 (FOXM1) and vascular endothelial growth factor (VEGF). These findings indicate that miR-184 promotes the apoptosis of endometrial epithelial cells in dairy goats by downregulating STC2 via the RAS/RAF/MEK/ERK pathway, and that it may also regulate the establishment of RE in dairy goats.

## 1. Introduction

Embryo implantation is a key step in mammalian reproduction [1]. The establishment of RE is one of the most important factors for embryo implantation [2]. Studies have shown that endometrial damage and dysplasia can lead to inadequate endometrial receptivity, which ultimately leads to pregnancy failure [3]. A series of complex dynamic changes in the endometrium eventually form a receptive endometrium (RE), and apoptosis of endometrial epithelial cells (EECs) is an essential step in this process [4,5]. Therefore, in order to better understand embryo implantation in dairy goats, it is necessary to study the apoptosis of EECs.

miRNAs are a class of endogenous noncoding RNAs which are generally involved in the regulation of posttranscriptional gene expression by indirect or direct inhibition of the translation of mRNA-bound 3′ untranslated region (UTR) [6]. miRNAs are involved in most biological processes in mammals, e.g., cell differentiation, proliferation and apoptosis [7]. Additionally, miRNAs regulate the endometrial function and embryo development during embryo implantation [8]. Our previous studies showed that some miRNA regulates EEC apoptosis and proliferation, and thus, participates in endometrial receptivity formation [9,10]. Additionally, studies have shown that miR-184 inhibits hepatocellular carcinoma cell proliferation and promotes hepatocellular carcinoma cell apoptosis by regulating the expression of AGO2 [11], and that miR-184 upregulates the expressions of P53 and P21 to inhibit the proliferation of human glioma and breast cancer cells [12]. Although many studies have found that miR-184 can regulate the proliferation and apoptosis of cancer cell, the effect of miR-184 on the proliferation and apoptosis of EECs in dairy goats remains unclear.

STC2 is a glycoprotein in the Stanniocalcin family. Studies have shown that STC2 regulates a series of biological processes through autocrine or paracrine signaling [13]. For example, STC2 promotes the proliferation of liver cancer cells and also inhibits adipogenic differentiation of human mesenchymal stem cells [14,15]. Furthermore, pregnancy-associated plasma protein-A2 (PAPP-A2) increases the expression of insulin-like growth factor 1 (IGF1) via the lytic IGF complex. At the same time, STC2 reduces the release of IGF1 by inhibiting the biological activity of PAPP-A2, thus participating in the regulation of human dental pulp cell differentiation [16,17]. Another study showed that STC2 activates the PI3K/AKT/Snail signaling pathway to promoted proliferation, promoted passage through the G1/S cell cycle transition, and inhibited apoptosis in carcinoma cells [18]. However, there is no research on the effect of STC2 on the endometrium of dairy goats.

In this study, miR-184 and STC2 were selected for the following three reasons: (1) Our previous sequencing results show that miR-184 levels in the endometrial receptive phase of the dairy goat were 31-fold higher than those in the prereceptive phase [19]; (2) 3′UTR of STC2 has a unique nucleotide sequence combined with the miR-184 seed sequence; (3) STC2 and miR-184 play an important role in cell proliferation, apoptosis and differentiation [11,15]. Moreover, we investigated the effect of miR-184 and STC2 on the EECs of dairy goats in vitro and the establishment of RE. On the basis of the present study, we concluded that miR-184 could target STC2 and inhibit its expression in EECs. miR-184 inhibited the RAS/RAF/MEK/ERK signaling pathway by inhibiting STC2, and inhibited the proliferation of EECs and promoted their apoptosis. Therefore, the regulation effect of miRNA and mRNA on endometrial receptivity of dairy goat was studied; the molecular mechanism of endometrial receptivity establishment of dairy goat at the molecular level was determined. This knowledge is of great significance for improving the success rate of embryo implantation in dairy goats.

## 2. Materials and Methods

### 2.1. Sample Collection and Cell Culture

In this study, all goats used for the experiments were Xinong Saanen dairy goats, raised according to the No. 5 proclamation of the Ministry of Agriculture, P. R. China. All procedures in our animal study were approved by the Animal Care and Use Committee of the Northwest A&F University (Yangling, China) (permit number: 17-347, data: 13 October 2017).

Endometrial tissue from the anterior wall of the uterine cavity was collected on day 5 (PE, *n* = 3) and day 15 (RE, *n* = 3) of pregnancy [19,20]. All endometrial tissues were immediately rinsed three times with phosphate-buffered saline (PBS), and then stored in PBS containing penicillin (100 U/mL) and streptomycin (100 mg/mL), for EECs isolation. Some tissue samples were immediately placed in liquid nitrogen and stored. The primary EECs were digested and purified using trypsin and differential centrifugation, and the cells were identified via immunofluorescence chemical tests as previously described [9]. The EECs were cultured in the Dulbecco’s Modified Eagle Medium/Nutrient Mixture F-12 (DMEM/F12), containing 10% fetal bovine serum (FBS), in a humidified incubator with 5% CO_2_ at 37 °C. HEK293T were cultured in high-glucose DMEM, containing 10% FBS, in a humidified incubator with 5% CO_2_ at 37 °C.

### 2.2. Cell Treatment and Total RNA Extraction

EECs were cultured in 6-well plates and grown to 50% confluence before transfection. Si-STC2, miR-184 mimic, negative control (NC), miR-184 inhibitor, and NC-inhibitor (NCH) were designed and synthesized by Ribobio (Guangzhou, China), and these were transfected into EECs using the Lip2000 liposome (Invitrogen, Carlsbad, CA, USA). The culture medium was changed 4 h after transfection and cultured for 24 h. Then, total RNA and tissue RNA were extracted following the RNAiso (TaKaRa, Dalian, China) instructions. The extracted RNA was analyzed for quality and concentration using an Epoch ultramicro microporous plate spectrophotometer (BioTek, Burlington, VT USA).

### 2.3. Luciferase Activity Assay

A bioinformatics analysis was performed on the binding sequence of STC2 and miR-184 using miRanda and Targetscan7.0 to identify the target gene of miR-184. In this experiment, 340 bp of STC2 3′UTR, containing the binding site sequence, was selected, and primers were designed. Not1 and Xho1 restriction sites were added to the upstream and downstream of the primers, respectively. The primer sequences are shown in Table 1. Then, 3′UTR of STC2 was cloned and inserted into psiCHECKTM2 vector (Promega, Madison, WI, USA) to construct STC2 wild-type (WT-STC2-3′UTR) double luciferase reporter vector, and the mutant plasmid with the mutant binding site was constructed successfully (MUT-STC2-3′UTR). All constructs were verified by sequencing. WT-STC2-3′UTR or MUT-STC2-3′UTR were cotransfected with miR-184 mimic or NC into 293T cells. After 36 h of culture, 293T cells were collected, and the double luciferase activity was evaluated. The specific protocol has been previously described [21]. Three replicates were used for each experiment.

### 2.4. RT-qPCR

RT-qPCR primers were designed using Primer 5.0 and synthesized by Sangon Biotech Company (Shanghai, China). All the primers for RT-qPCR are shown in Table 1. Total RNA was reverse transcribed into cDNA using the Prime Script RT reagent Kit with gDNA Eraser (TaKaRa, Dalian, China) according to the manufacturer’s instructions, and an Epoch ultramicro microporous plate spectrophotometer was used to detect cDNA quality and concentration. The RT-qPCR reaction mixture was prepared following the instructions of SYBR Green PCR Master Mix (TaKaRa, Dalian, China). CFX Connect (Bio-Rad, Burlington, VT, USA) was used to perform RT-qPCR; the reaction procedure was as previously described [22]. β-actin was used as a reference for genes and U6 as a reference for miR-184. Three replicates were performed for each experiment.

### 2.5. Protein Extraction and Western Blot (WB) Analysis

After 48 h of EEC treatment, the original culture medium was discarded and cells were washed with PBS three times, placed on ice, and mixed with RIPA cell lysate (Applygen Technologies Inc., Beijing, China), containing protease and phosphatase inhibitors (Roche, Basel, Switzerland). The protein extraction method and WB analysis have been described previously [9,23]. All test antibodies are shown in Table 2. The blots were visualized using enhanced chemiluminescence (Advansta, Menlo Park, CA, USA), and related data were analyzed using ImageJ processing software; each experiment was performed in triplicate.

### 2.6. Vector Construction

For the construction of STC2 overexpression vector, the coding domain sequence (CDS) of goat *STC2* in NCBI was used as a template to design primers, and the restriction enzyme digestion sites, Kpn1h and Xho1, were added to the upstream and downstream of the primers, respectively. Also, 2–3 protective bases were added before the restriction enzyme digestion sites; the primer sequences are shown in Table 1. The CDS of *STC2* was cloned and inserted in the psiDNA3.1(+) vector (Promega, Madison, WI, USA). The empty vector with no STC2 sequence was used as the NC.

### 2.7. Cell Proliferation Analysis

The CCK-8 (ZETA, San Francisco, CA, USA) was used to detect the viability of EECs in vitro. EECs were inoculated in 96-well plates and grown to 50% confluence before transfection. The steps are as described previously [24]. The optical density was measured at 450 nm using an Epoch ultramicro microporous plate spectrophotometer (BioTek, Burlington, VT, USA); each experiment was performed in triplicate. The EDU (Ribobio, Guangzhou, China) assay was used to measure the proliferation of EECs following the manufacturer’s instructions. The steps are as described previously [25]. We used Olympus IX73 microscope (Olympus, Tokyo, Japan) to visualize and count EDU cells. Each experiment was performed in sextuplicate.

### 2.8. Cell Apoptosis Assay

The flow cytometry method (FCM) and the Annexin V-FITC/PI apoptosis kit (Liankebio, Hangzhou, China) were used to detect EECs apoptosis, according to the manufacturer’s instructions. The reaction was carried out as per a procedure described previously [26].

### 2.9. Statistical Analysis

All data were analyzed using SPSS17.0 (SPSS Inc., Chicago, IL, USA). One-way ANOVA was used to compare the differences, and the least significant difference method was used for further analysis. All data were expressed as the mean ± standard error. The differences were considered significant when *p* < 0.05 and extremely significant when *p* < 0.01.

## 3. Results

### 3.1. Differential Expression of miR-184 and STC2 in PE and RE Endometrial Tissues

**Figure 1:**
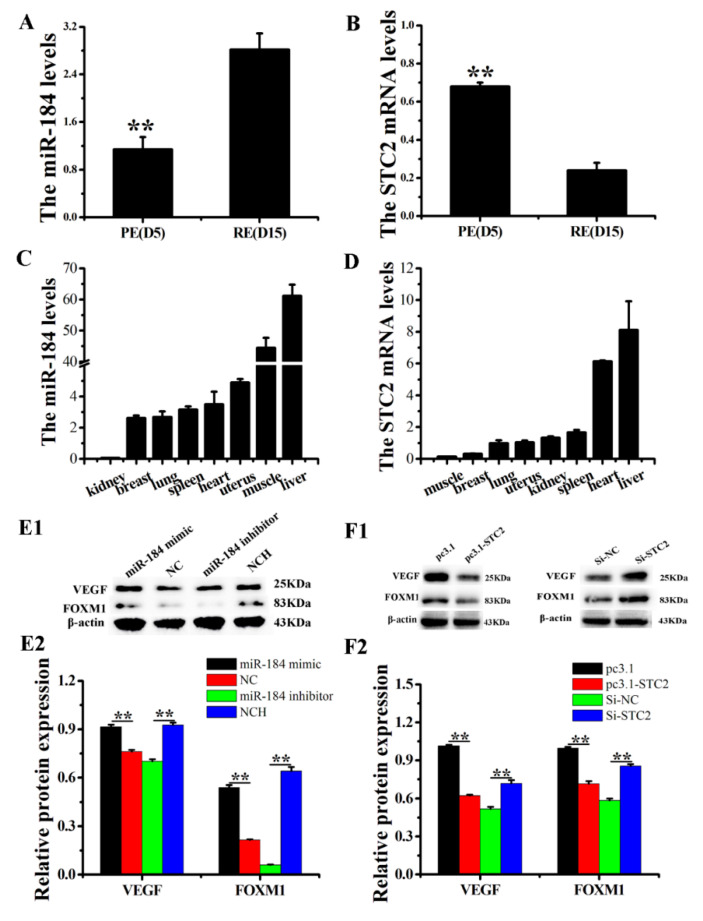
The expression levels of miR-184 and STC2 in dairy goats. (**A**) The expression of miR-184 in PE and RE in dairy goats. (**B**) The expression of STC2 in PE and RE in dairy goats. (**C**) The expression of miR-184 in various tissues of dairy goats. (**D**) The expression of STC2 in various tissues of dairy goats. After transfection with miR-184 (**E1**,**E2**) or STC2 (**F1**,**F2**), WB analyzed the protein expression levels of VEGF and FOXM1. Stem-loop RT-qPCR was used to detect the expression of miR-184, which was standardized to that of U6. RT-qPCR was used to detect the expression of STC2, which was standardized to that of β-actin. The WB was used to measure protein expression and the optical density method was standardized to the density of β-actin in the same lane. Data is expressed as the “mean ± SEM” of three values, ** *p* < 0.01; PE, pre-receptive endometrium; RE, receptive endometrium; SEM, standard error of the mean.

In our previous studies, the results showed that the expression of miR-184 in RE increased by 31 times compared to that in PE [19]. In this study, the RT-qPCR results show that the expression of miR-184 in RE was significantly increased compared to that in PE (Figure 1A), which was consistent with the previous sequencing results. Furthermore, the expression of STC2 mRNA in RE was significantly reduced compared to that in PE (Figure 1B), which was in contrast to the trend of miR-184 expression. Furthermore, miR-184 and STC2 were widely expressed in the heart, liver, spleen, lungs, kidneys, muscles, uterus, and breasts of dairy goats (Figure 1C,D). The results indicated that the expression levels of miR-184 were significantly increased by the miR-184 mimic and reduced by the miR-184 inhibitor (Figure S1A, *p* < 0.01).

### 3.2. miR-184 and STC2 Regulate the Expression of Some RE Markers in Dairy Goats

To further verify the regulatory effect of miR-184 and STC2 on the establishment of RE, we tested the expression of RE marker genes, VEGF and FOXM1. miR-184 mimic significantly upregulated VEGF and FOXM levels, while miR-184 inhibitors significantly downregulated their levels (Figure 1(E1,E2), *p* < 0.01). Additionally, overexpressing STC2 decreased the levels of VEGF and FOXM significantly, while knock-down of STC2 increased their levels significantly (Figure 1(F1,F2), *p* < 0.01).

### 3.3. STC2 Is the Target of miR-184

**Figure 2:**
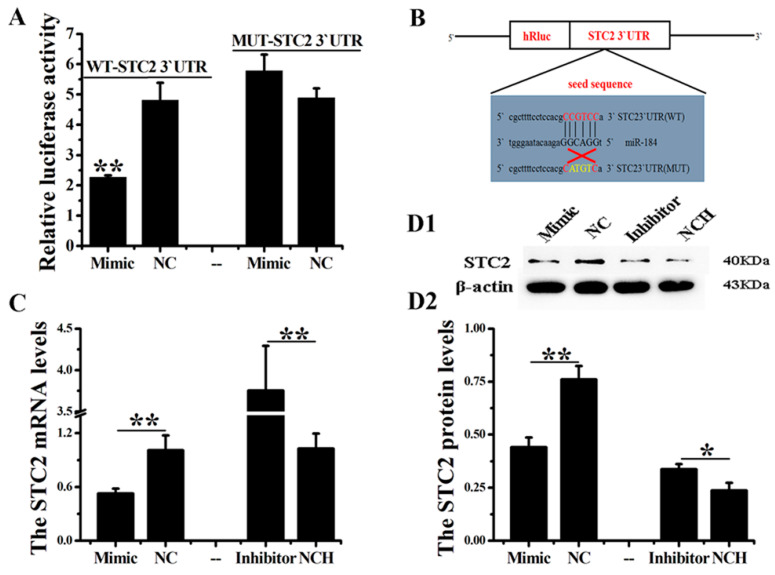
*STC2* is the target of miR-184. (**A**) Schematic diagram of dual-luciferase reporter design for the wild-type (WT-STC2) or mutant (MUT-STC2). The red sequence represents the “seed sequence” of miR-184, and the yellow sequence represents the mutant sequence. (**B**) Double luciferase activity was detected after 293T cells were cotransfected with WT-STC2 or MUT-STC2 and miR-184 or NC (negative control). miR-184 upregulates the expression of STC2 at mRNA (**C**) and protein (**D1**,**D2**) levels. STC2 expression was detected using RT-qPCR and normalized to β-actin. WB analysis was used to measure expression at the protein level. The optical density method was standardized to the density of β-actin in the same lane. Data is expressed as the “mean ± SEM” of three values, ** *p* < 0.01; * *p* < 0.05. WB, western blot; SEM, standard error of the mean.

In this study, STC2 was predicted as the target of miR-184 using two publicly available programs (Targetscan 7.0 and miRanda). We successfully predicted the target binding sequence of miR-184 and STC2. To confirm this prediction, we constructed a dual-luciferase reporter vector (Figure 2A). The dual-luciferase test results show that miR-184 significantly downregulates the luciferase activity of WT-STC2, but has no significant effect on MUT-STC2 (Figure 2B, *p* < 0.01). Additionally, miR-184 significantly reduces the expression of STC2 mRNA in EECs (Figure 2C, *p* < 0.01), and decreases the expression of STC2 in EECs (Figure 2(D1,D2), *p* < 0.01). The results show that STC2 is the target of miR-184, and miR-184 inhibits the expression of STC2 at the mRNA and protein levels in EECs by binding to the 3′UTR of STC2.

### 3.4. miR-184 Inhibits the Proliferation and Promotes EECs Apoptosis

**Figure 3:**
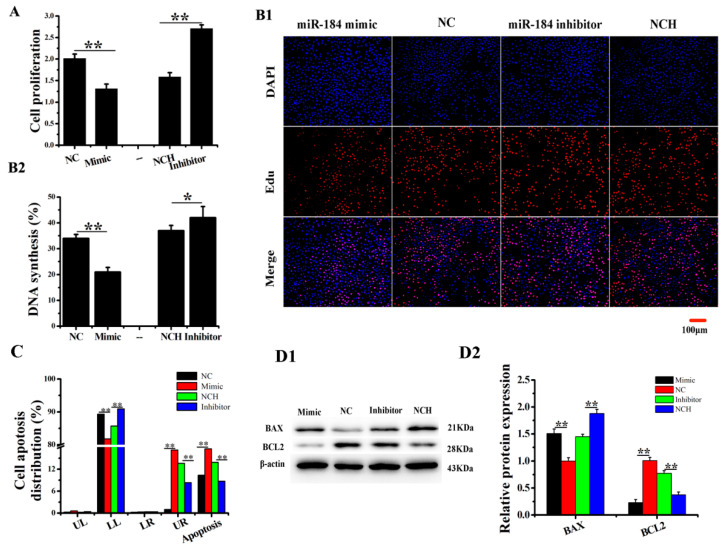
miR-184 promoted EECs apoptosis in vitro. (**A**) CCK-8 was used to detect cell viability. (**B1**,**B2**) Cell proliferation indices were assessed after treatment with EDU. The scale bar is 100 μm. (**C**) FCM was used to detect apoptosis. (**D1**,**D2**) The WB analysis revealed the expression of BCL2 and BAX in EECs transfected with miR-184 mimic, NC, miR-184 inhibitor, or NCH. The optical density method was normalized to the density of β-actin in the same lane. Data is expressed as the “mean ± SEM” of three values, ** *p* < 0.01; * *p* < 0.05. CCK-8, cell counting kit-8; WB, western blot; EDU, 5-Ethynyl-2′-deoxyuridine; FCM, flow cytometry method; EECs, endometrial epithelial cell; SEM, standard error of the mean; NC, negative control; NCH, NC-inhibitor. “UL”, “LL”, “LR” and “UR” represented, respectively, the CEEC of mechanical damage, normal, early apoptosis, and late apoptosis.

To verify the effect of miR-184 on EECs, we used CCK8 and EDU and analyzed cell proliferation. The CCK-8 assay results show that miR-184 mimic significantly reduces cell viability compared to NC, while miR-184 inhibitor significantly increases it compared to NCH (Figure 3A). Also, the EDU assay results show that miR-184 mimic significantly inhibits the proliferation of EECs compared to NC, but that miR-184 inhibitor significantly promotes that of EECs compared to NCH (Figure 3(B1,B2)). Moreover, the apoptosis results show that miR-184 mimic promotes EECs apoptosis, while miR-184 inhibitor inhibits it (Figure 3C and Figure S2A–D). We also detected the expression of apoptosis-related genes, BCL2 and BAX. The results show that miR-184 mimic significantly reduces the expression of BCL2 and increases the expression of BAX, but that miR-184 inhibitor significantly increases the expression of BCL2 and reduces the expression of BAX (Figure 3(D1,D2), *p* < 0.01). The results of the aforementioned experiments indicate that miR-184 promotes the proliferation of EECs and inhibits their apoptosis.

### 3.5. The Effect of STC2 on EECs

**Figure 4:**
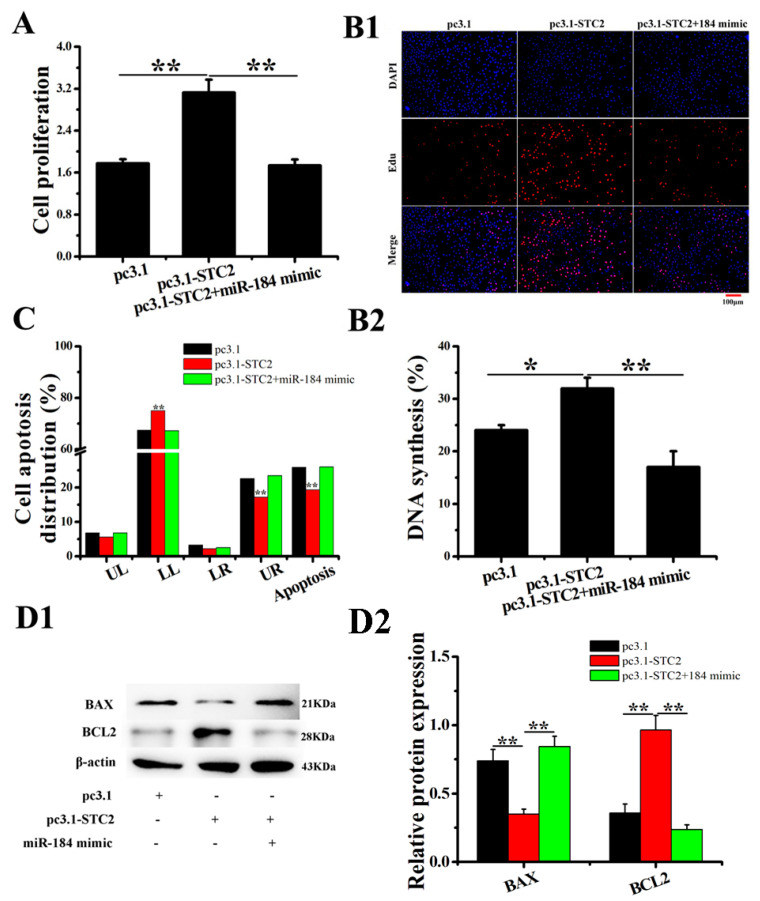
Overexpression of STC2 inhibited the EECs apoptosis in vitro. (**A**) pc3.1-STC2 and/or miR-184 mimics were transfected into EECs, and CCK-8 was used to detect cell viability. (**B1**,**B2**) Cell proliferation indices were assessed after treatment with EDU. The scale bar is 100 μm. (**C**) FCM was used to detect apoptosis. (**D1**,**D2**) The WB analysis revealed the expression of BCL2 and BAX in EECs. The optical density method was normalized to the density of β-actin in the same lane. Data are expressed as the “mean ± SEM” of three values, ** *p* < 0.01; * *p* < 0.05. CCK-8, cell counting kit-8; WB, western blot; EDU, 5-Ethynyl-2′-deoxyuridine; FCM, flow cytometry method; EECs, endometrial epithelial cell; SEM, standard error of the mean.

In order to verify the regulatory effect of STC2 on EECs, an STC2 overexpression vector (pc3.1-STC2) was successfully constructed. After transfecting EECs with pc3.1-STC2, the mRNA and protein expression levels of STC2 increased significantly, whereas the miR-184 mimic weakened the expression of STC2, indicating that pc3.1-STC2 could be used in subsequent experiments (Figure S3A–C). The EDU and CCK-8 assay results show that STC2 significantly promotes the proliferation of EECs, while miR-184 reduces this effect of STC2 (Figure 4(A,B1,B2)). Furthermore, the results of the apoptosis test show that STC2 significantly inhibits EECs apoptosis, while miR-184 weakens this effect of STC2 (Figure 4C and Figure S4A–C). The WB analysis results show that STC2 significantly downregulates BAX expression and upregulates BCL2 expression (Figure 4(D1,D2), *p* < 0.01).

**Figure 5:**
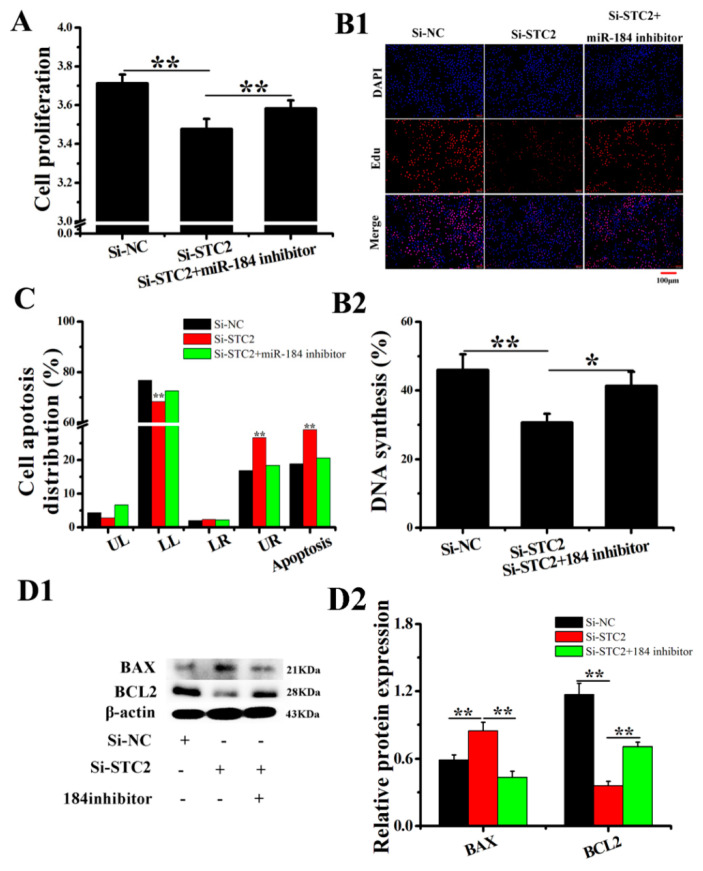
Interference with STC2 promoted EEC apoptosis in vitro. (**A**) Si-STC2 and/or miR-184 inhibitor were transfected into EECs, and the CCK-8 was used to detect cell viability. (**B1**,**B2**) Cell proliferation indices were assessed after treatment with EDU. The scale bar was 100 μm. (**C**) FCM was used to detect apoptosis. (**D1**,**D2**)The WB analysis revealed the expression of BCL2 and BAX in EECs. The optical density method was normalized to the density of β-actin in the same lane. Data is expressed as the “mean ± SEM” of three values, ** *p* < 0.01; * *p* < 0.05. CCK-8, cell counting kit-8; WB, western blot; EDU, 5-Ethynyl-2′-deoxyuridine; FCM, flow cytometry method; EEC, endometrial epithelial cell; SEM, standard error of the mean.

The Si-STC2 designed and synthesized by Ribobio Company was transfected into EECs, which significantly reduced the mRNA and protein expression levels of STC2 (Figure S3D–F). Additionally, the EDU and CCK8 assay results show that Si-STC2 significantly inhibits the proliferation of EECs, while miR-184 inhibitor reduces this effect of Si-STC2 (Figure 5(A,B1,B2)). MiR-184 inhibitors reduced the expression of miR-184 and weakened the inhibitory effect of miR-184 on STC2, so the Si-STC2+ miR-184 inhibitor group significantly promoted EEC proliferation compared with the Si-STC2 group. Meanwhile, Si-STC2 significantly promoted the apoptosis of EECs, and the miR-184 inhibitor weakened this effect of Si-STC2 (Figure 5C and Figure S4D–F). Next, Si-STC2 significantly downregulated the expression of BCL2 and upregulated the expression of BAX (Figure 5(D1,D2), *p* < 0.01). The results show that STC2 promotes the proliferation and inhibits the apoptosis of EECs.

### 3.6. miR-184 Regulates the RAS/RAF/MEK/ERK Pathway Through the STC2 in EECs

**Figure 6:**
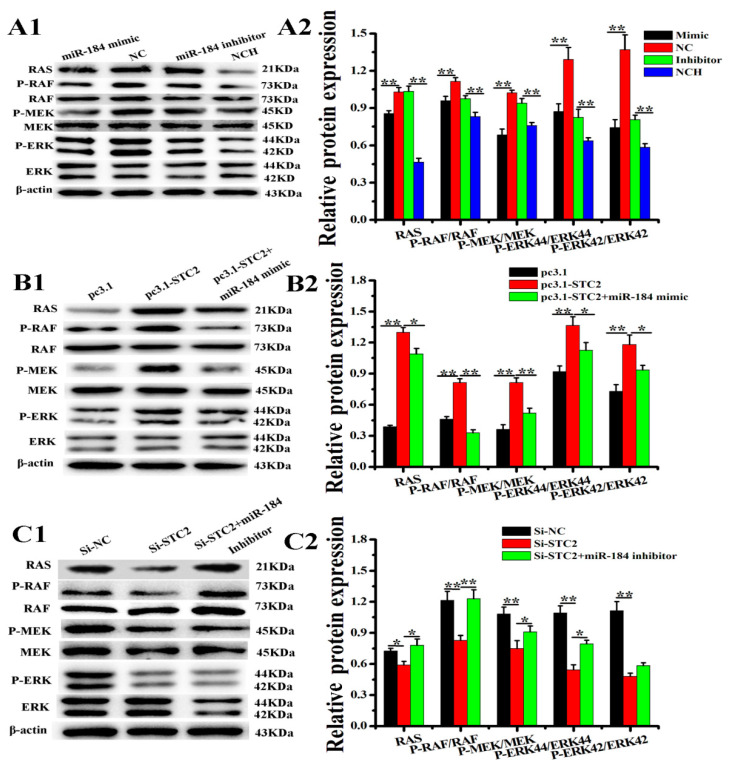
Effects of miR-184 and STC2 on the RAS/RAF/MEK/ERK pathway. (**A1**,**A2**) WB analysis of RAS, p-RAF, RAF, p-MEK, MEK, p-ERK, and ERK regulated by miR-184. (**B1**,**B2**) After transfection of EECs with pc3.1-STC2 or/and miR-184 mimic, WB was used to detect the expression levels of RAS, p-RAF, RAF, p-MEK, MEK, p-ERK, and ERK at the protein level in EECs. (**C1**,**C2**) After transfection of EECs with Si-STC2 or/and miR-184 inhibitor, WB was used to detect the expression levels of RAS, p-RAF, RAF, p-MEK, MEK, p-ERK, and ERK at the protein level in EECs. The optical density method was normalized to the density of β-actin in the same lane. Data is expressed as the “mean ± SEM” of three values, ** *p* < 0.01; * *p* < 0.05. WB, western blot; SEM, standard error of the mean.

To explore the effects of miR-184 and STC2 on EECs survival further, we detected the expression levels of proteins involved in the RAS/RAF/MEK/ERK signaling pathway. The WB analysis results show that miR-184 significantly reduces the expression levels of RAS, P-RAF/RAF, P-MEK/MEK, and P-ERK/ERK, but that the miR-184 inhibitor significantly increases the expression levels of RAS, P-RAF/RAF, P-MEK/MEK, and P-ERK/ERK (Figure 6(A1,A2), *p* < 0.01). Additionally, pc3.1-STC2 significantly increases the expression levels of RAS, P-RAF/RAF, P-MEK/MEK, and P-ERK/ERK (Figure 6(B1,B2), *p* < 0.01), and STC2 knockdown significantly reduces the expression levels of RAS, P-RAF/RAF, P-MEK/MEK, and P-ERK/ERK (Figure 6(C1,C2), *p* < 0.01). The results show that miR-184 inhibits the RAS/RAF//MEK/ERK signaling pathway by targeting STC2.

## 4. Discussion

In previous studies, we reported, for the first time, that miRNAs were differentially expressed in the PE and RE of dairy goats [19]. Furthermore, we verified that miR-449a, miR-181a, and miR-26a play important roles during the establishment of endometrial receptivity in dairy goats [9,10,27]. In the current study, the RT-qPCR results show that miR-184 expression in dairy goats RE is significantly higher than that in PE, which was consistent with our previous sequencing results [19]. Additionally, studies have shown that miR-184 promotes the apoptosis of trophoblast cells and inhibits their proliferation, which plays an important role in maintaining pregnancy [28]. The aforementioned findings indicate that miR-184 may play an important role in the establishment of RE in dairy goats.

The establishment of RE is a dynamic and complex process, and cell proliferation and apoptosis play an important role [29]. Our previous results have shown that miR-34a/c and miR-181a participate in the establishment of endometrial receptivity by regulating EECs apoptosis in dairy goats [27,30]. Many studies have shown that miR-184 can be used as an oncogene or tumor suppressor. For example, miR-184 inhibits the proliferation and invasion of human glioma and breast cancer cells [12], and blocks the growth and survival of nasopharyngeal carcinoma cells by directly targeting *BCL2* and C-*MYC19* [31]. Many studies have shown that BAX is pro-apoptotic and BCl2 is anti-apoptotic [32,33]. In the current study, WB analysis showed that miR-184 upregulated the expression of BAX in EECs of dairy goats and downregulated the expression of BCL2. Furthermore, our results show that miR-184 promotes EECs apoptosis.

Generally, miRNA binds to the target gene 3′UTR seed sequence to induce degradation of the target mRNA or inhibit its translation, thus participating in various biological processes, such as cell proliferation, apoptosis and differentiation [34]. For example, miR-532 induces apoptosis of human nucleus pulposus cells by inhibiting its target, *Bcl-9* [35]. Here, we found that there was a seed sequence between *STC2* and miR-184, and that miR-184 downregulated the dual-luciferase activity of MUT-STC2, thereby downregulating the expression of STC2 at mRNA and protein levels in dairy goat EECs. These results indicate that *STC2* is the target of miR-184 in dairy goat EECs.

STC2 plays an important role in the regulation of the proliferation and apoptosis of cancer cells [18,36]. STC2 interference inhibits the proliferation of ovarian cancer cells, while its overexpression promotes proliferation [37]. Here, apoptosis and proliferation assay results show that STC2 promotes the proliferation of EECs in dairy goats and inhibits their apoptosis. These results also show that *STC2* is the target of miR-184, and that miR-184 might regulate the EECs apoptosis in dairy goats through STC2.

Studies have shown that VEGF is an endometrial receptive biomarker [38,39], and that FOXM1 upregulates the expression of VEGF [22]. FOXM1 plays an important role in the establishment of RE in mice [40]. Additionally, the results of the current study show that miR-184 upregulates the expression of VEGF and FOXM1 in the EECs of dairy goats in vitro. Moreover, WB analysis showed that the overexpression of STC2 reduced the expression of VEGF and FOXM1, while interference with STC2 increased the expression of VEGF and FOXM1. Therefore, we concluded that miR-184 and STC2 participate in the establishment of RE by regulating VEGF and FOXM1 in vitro. However, in vivo, the development of miR-184 and STC2 in endometrial receptivity needs further study.

Research shows that the RAS/RAF/MEK/ERK pathway is key for cell proliferation and apoptosis [41,42]. Moreover, studies have shown that the RAS/RAF/MEK/ERK pathway regulates the apoptosis of EECs [30]. Studies have also found that STC2 regulates cell proliferation and apoptosis in colorectal cancer cells by activating the RAS/RAF/MEK/ERK pathway [36]. Therefore, in this experiment, we tested the expression of RAS/RAF/MEK/ERK. The results show that STC2 activates the RAS/RAF/MEK/ERK pathway, while miR-184 inhibits it. In summary, miR-184 and STC2 participate in the regulation of the proliferation and apoptosis of EECs in dairy goats by regulating the RAS/RAF/MEK/ERK pathway.

## 5. Conclusions

From these findings, we conclude that miR-184 promotes the apoptosis of EECs in dairy goats by downregulating STC2 via the RAS/RAF/MEK/ERK pathway, and that miR-184 contributes to the establishment of endometrial receptivity. Additionally, miR-184 may be a biomarker of RE in dairy goats. This study may contribute to our knowledge of the regulation molecular mechanism of embryo attachment in dairy goats. It may also be important in improving the reproduction rate of dairy goats.

## Figures and Tables

**Table 1:** All primers used in this study.

Gene	GenBank Accession No.	Primer Sequence (5′–3′)
STC2 (qPCR)	XM_005694539.3	F: CGGAAGTGTCCAGCCATCAAGG
		R:GCAGCAGTCACACACAGTCA
STC2 (Check2)	/	F: CGCTCGAGTCGGGAGACCGGCCGAGG
		R: ATGCGGCCGCTCGCTGCCCAGGGAGCCT
STC2 (pcDNA3.1)	/	F: CAG*GGTACC*ATGTGTGCCGAGCGGCTG
		R:CG*CTCGAG*TCACCTCCGGATATCGGAATACTCAGACTGTTC
β-actin	XM_018039831.1	F: GATCTGGCACCACACCTTCT
		R: GGGTCATCTTCTCACGGTTG
U6	/	F: CTCGCTTCGGCAGCACA
		R: AACGCTTCACGAATTTGCGT
miR-184-Loop	/	gtcgtatccagtgcagggtccgaggtattcgcactggatacgacACCCTTAT
miR-184	/	gcgcgcTGGACGGAGAACTG
Reverse Primer	/	GTGCAGGGTCCGAGGT

Note: The underlined characters are the restriction sites of xhoI and notI which were used to construct psiCHECK2. Italics and underline indicates the restriction sites of KpnI and xhoІ which were used to construct pcDNA3.1.

**Table 2:** Antibody information.

Name	Manufacturer	Product Number
STC2	Gene Tex, America	GTX82231
β-actin	Beyotime, Shanghai, China	AA128
BAX	Beyotime, Shanghai, China	AB026
BCL2	Beyotime, Shanghai, China	AB112
VEGF	Boster, Wnhan, China	BA0407
FOXM1	SAB, United States	#32671
RAS	Gene Tex, America	GTX132480
RAF	BBI, Shanghai, China	D220484
P-RAF (Ser642)	BBI, Shanghai, China	D151385
MEK1	Abways, Shanghai, China	CY5168
P-MEK1 (Ser298)	Abways, Shanghai, China	CY5277
ERK	Cell Signaling, America	#9101
P-ERK (Thr202/Tyr204)	Cell Signaling, America	#9102
HRP-labeled goat anti-rabbit IgG (H + L)	Beyotime, Shanghai, China	A0208
HRP-labeled goat anti-mouse IgG (H + L)	Beyotime, Shanghai, China	A0216

## Supplementary Materials

The following are available online at https://www.mdpi.com/2073-4425/11/9/1052/s1, Figure S1: Efficiency of miR-184 transfection into EECs, Figure S2: MiR-184 promoted apoptosis of EECs, Figure S3: The transfection efficiency of Si-STC2 and pc3.1-STC2 into EECs, Figure S4: STC2 inhibited apoptosis of EECs.
