# Supplementary figures and images for: MiR-184 Combined with STC2 Promotes Endometrial Epithelial Cell Apoptosis in Dairy Goats via RAS/RAF/MEK/ERK Pathway
Source: Genes (Basel). 2020 Sep 7;11(9):1052. doi: 10.3390/genes11091052 (PMC7565287; doi:10.3390/genes11091052)

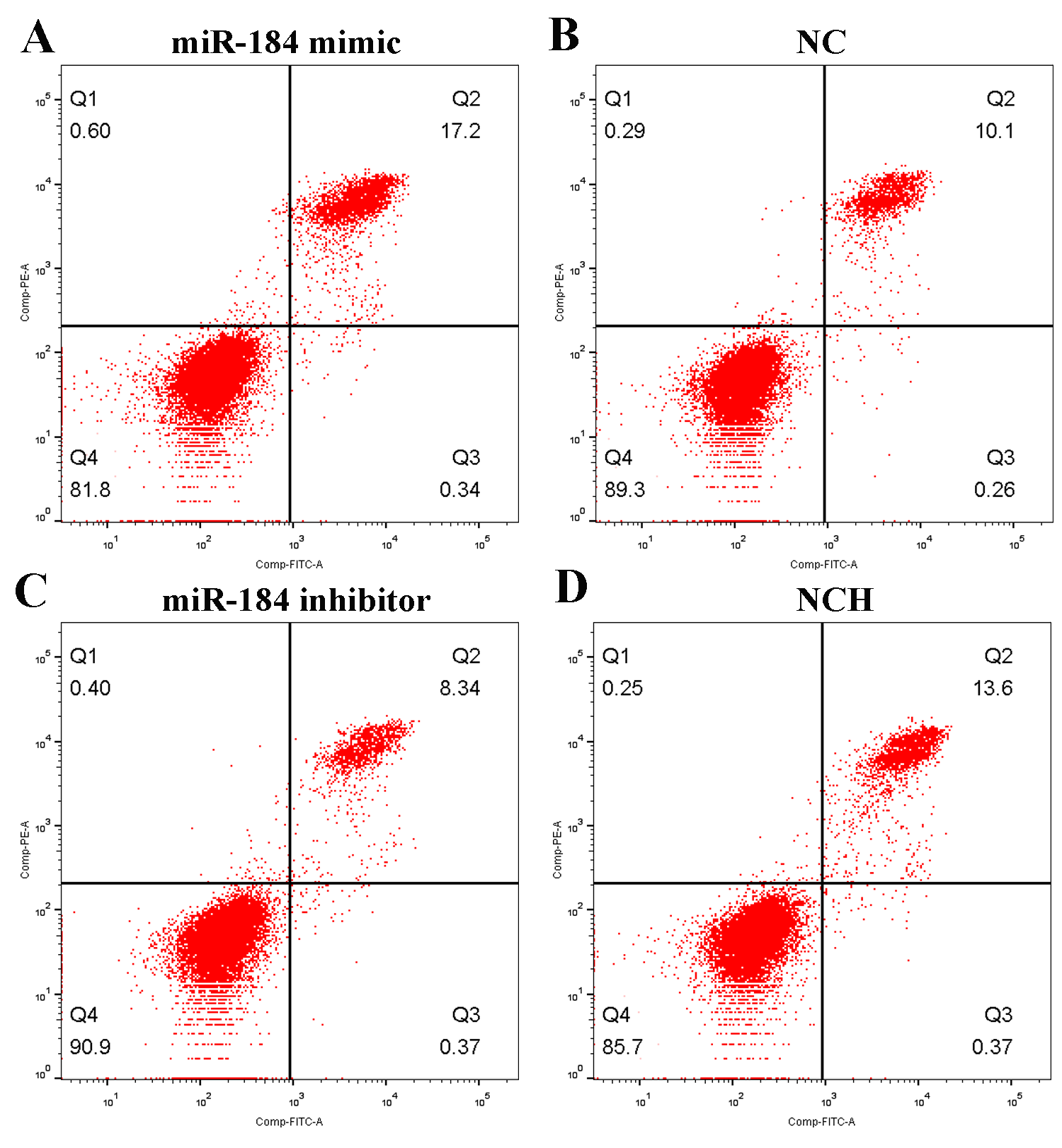

Supplement: Supplementary file 1 [file genes-11-01052-s001.zip › Figure.S1-4/Figure.S2.tif]

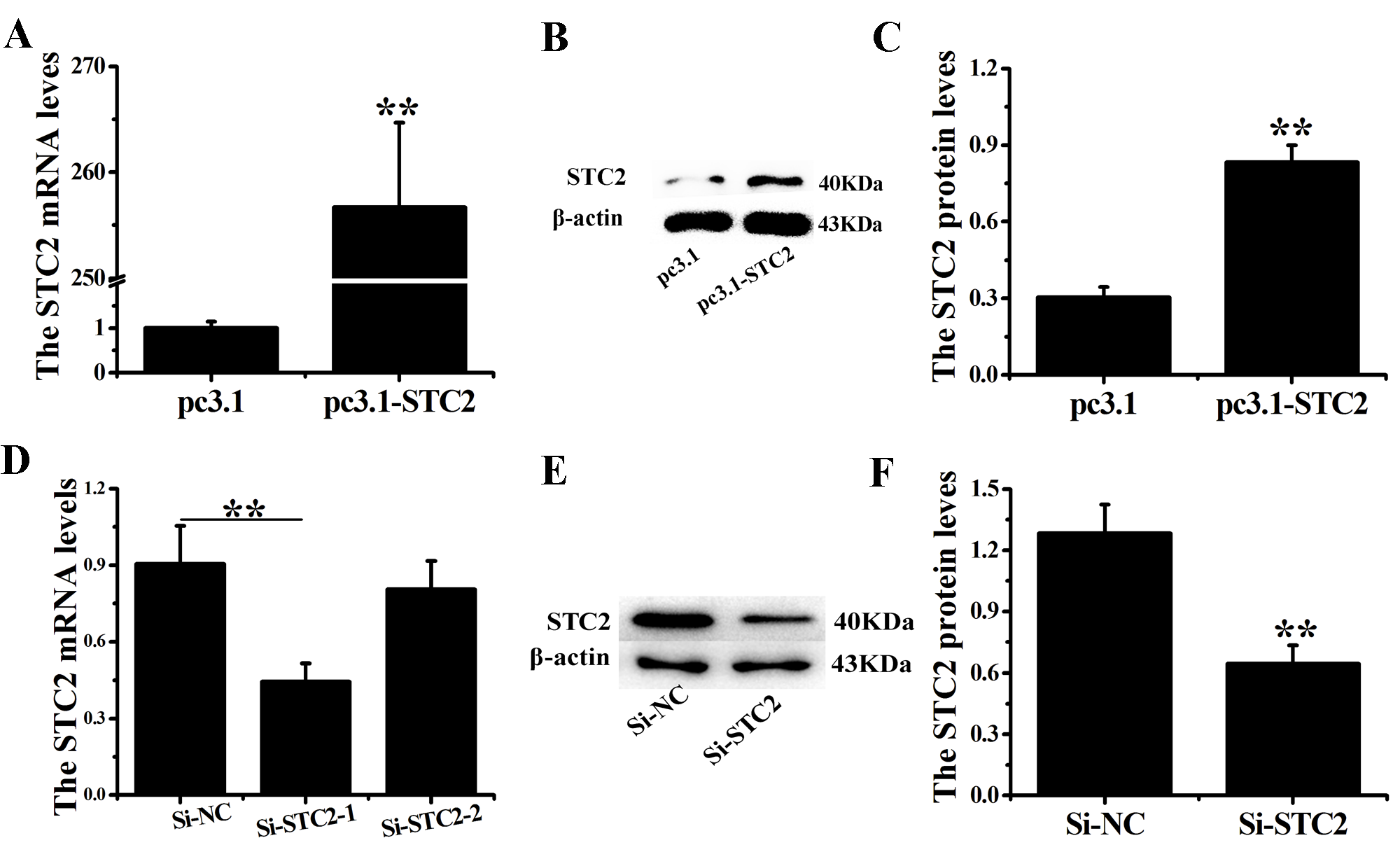

Supplement: Supplementary file 1 [file genes-11-01052-s001.zip › Figure.S1-4/Figure.S3.tif]

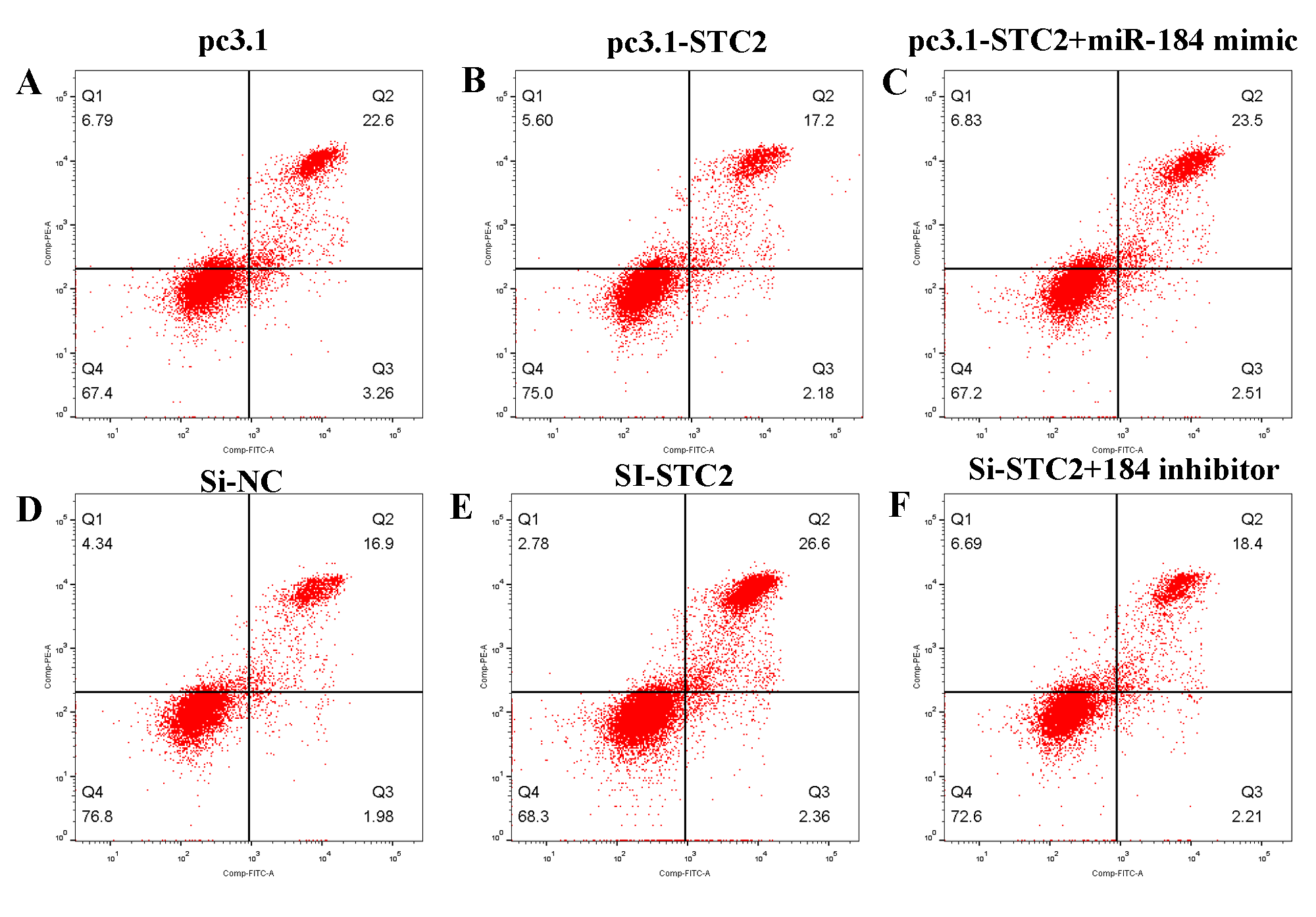

Supplement: Supplementary file 1 [file genes-11-01052-s001.zip › Figure.S1-4/Figure.S4.tif]
